# Ketogenic diets and metabolic dysfunction-associated steatotic liver disease: a literature review

**DOI:** 10.1186/s13578-025-01494-8

**Published:** 2025-12-15

**Authors:** Kexin Sun, Weitian Li, Yunan Chen, Edmund Anthony Severn Nelson, Xu Chen, Lai Ling Hui

**Affiliations:** 1 Department of Pediatrics, The Chinese University of Hong Kong, Hong Kong, Hong Kong SAR China; 2 School of Hotel and Tourism Management, The Hong Kong Polytechnic University, Hung Hum, Hong Kong SAR China; 3 Department of Food Science and Nutrition, The Hong Kong Polytechnic University, Hung Hom, Hong Kong SAR China; 4 School of Medicine, The Chinese University of Hong Kong, Shenzhen, Guangdong 518172 China

**Keywords:** Ketogenic diets, Metabolic dysfunction-associated steatotic liver disease, Liver steatosis, Lipid profile, Inflammation, Gut microbiota

## Abstract

Metabolic dysfunction-associated steatotic liver disease (MASLD) is estimated to affect over 30% of the global population with a rising trend, posing significant healthcare burden due to its progression and increased risk of related metabolic diseases. Dietary intervention plays an important role in the prevention and management of MASLD. Ketogenic diets represent a range of high-fat, moderate-protein, very low-carbohydrate (< 20–50 g/day) diets that induce nutritional ketosis. These diets have been proposed to benefit patients with MASLD by promoting weight loss, reducing inflammation and insulin resistance through different pathways. This review summarized the current findings on the outcomes of ketogenic diets on patients with MASLD regarding the liver, plasma lipid profile, systemic inflammation and gut microbiota. Studies showed that short- to medium- term ketogenic diets, with or without calorie restriction, are able to lower plasma triglycerides and ameliorate hepatic steatosis, steatohepatitis and fibrosis in MASLD. In particular, studies found ketogenic diets may be more effective in alleviating hepatic steatosis in short time periods than calorie-matched, high-carbohydrate, low-fat diets. Evidence on the impact on plasma high-density lipoprotein cholesterol (HDL-c) and low-density lipoprotein cholesterol (LDL-c) was mixed. Clinical trials investigating the effects on different markers of systemic inflammation and the composition of gut microbiota among patients with MASLD were scarce. To better understand the role of ketogenic diets in MASLD management, longer-term, well-controlled trials are warranted to clarify their potential benefits and risks, and whether they are varied by types of fats. Appropriate and sustainable formulations of ketogenic diets that maximize benefits and minimize side effects remain to be determined.

## Introduction

Metabolic dysfunction-associated steatotic liver disease (MASLD), previously referred to as non-alcoholic fatty liver disease (NAFLD), represents the latest terminology for steatotic liver disease linked to metabolic syndrome [1]. The new definition highlights the critical role of metabolic dysfunction in the etiology and pathogenesis. It has been shown that there is minimal difference between the patient populations of the two definitions, so research findings on NAFLD also apply to the new definition [2] and we used “MASLD” throughout this review.

MASLD has become the leading cause of chronic liver disease. A recent meta-analysis estimated the global prevalence of MASLD at 32.4% with a rising trend [3], which presents a considerable challenge for healthcare systems worldwide. MASLD represents a continuum of chronic hepatic disorders characterized by excessive triglyceride accumulation within the cytoplasm of hepatocytes. The disease spectrum begins with isolated hepatic steatosis, termed metabolic dysfunction-associated steatotic liver (MASL). It may subsequently involve lobular or portal inflammation and hepatocyte injury (ballooning), referred to as metabolic dysfunction-associated steatohepatitis (MASH), and can further progress to hepatic fibrosis, cirrhosis, and/or hepatocellular carcinoma [4]. A systematic review of observational studies revealed that 31% of individuals with MASL progressed to MASH within a median of 4.7 years [5]. Among patients with MASH, 10% developed end-stage liver disease within a median of 13 years, and the proportion rose to 25% for those with advanced fibrosis at baseline [6]. Around 7% of patients with MASLD suffered from liver-related mortality within a similar timeframe [7]. MASLD’s impact may extend beyond the liver. Patients with MASLD face an elevated risk of cardiovascular disease (CVD), obstructive sleep apnea, osteoporosis, chronic kidney disease, and various extrahepatic malignancies [8]. CVD is the principal cause of death among patients with MASLD [9], accounting for > 40% mortality, while malignant and non-malignant liver diseases caused < 10% [10]. These statistics underscore the needs to tackle modifiable risk factors of MASLD to prevent its development and halt or reverse its progression.

Previously the two-hit model suggested a simplistic sequence of lipid accumulation followed by inflammation in MASLD pathogenesis, but the updated multiple-hit hypothesis highlighted the simultaneous and interdependent actions of various factors that contribute to the disease and its progression [11]. Central to this model is insulin resistance, which promotes hepatic fat accumulation by increasing de novo lipogenesis and the influx of free fatty acids into the liver from the adipose tissue due to impaired inhibition of lipolysis [12]. This leads to lipotoxicity, where toxic lipid intermediates trigger oxidative stress, mitochondrial dysfunction and endoplasmic reticulum stress, further exacerbating hepatocellular damage [13]. The dysfunctional adipose tissue secrets pro-inflammatory cytokines such as tumor necrosis factor-alpha (TNF-α) and interleukin (IL)-6, amplifying systemic and local inflammation [14]. Additionally, altered gut microbiota enhances intestinal permeability, which increases the release of endotoxins and pro-inflammatory cytokines, contributing to chronic hepatic inflammation via the gut-liver axis [15]. Genetic predisposition [16] and epigenetic modifications [17] further modulate the susceptibility to MASLD and influence lipid metabolism, inflammatory responses and fibrogenesis. Collectively, these parallel and interacting hits create a dynamic and multifactorial process that drives MASLD development.

Despite the recent approval of the first medication, Resmetirom, for treatment of MASH with fibrosis [18], lifestyle intervention remains vital in MASLD management [19, 20]. In recent years, ketogenic diets, rich in fat and low in carbohydrates, have gained significant attention. The diets' primary goal is to induce ketosis, a metabolic state where the body relies on fats instead of glucose for energy due to limited carbohydrate availability, elevating the levels of ketone bodies in the blood [21]. Acetoacetate is the major ketone body produced in the mitochondria of the liver. It is then converted to beta-hydroxybutyrate (βHB), the major circulating ketone, and acetone, which is volatile and can be eliminated via respiration by the lungs, causing fruity breath [22]. Nutritional ketosis is defined as having a minimum serum ketone level of 0.5 mM [23] and the maximum can be 7-8 mM, unlike in diabetic ketoacidosis where the level can exceed 20 mM causing disrupted blood pH [22]. The potential benefits of ketogenic diets in managing obesity-related metabolic diseases like MASLD and its comorbidities have been an important research focus.

This review aims to summarize from published literature on the use of ketogenic diets on MASLD, including their benefits and risks. Since the lack of protein and choline in the typical ketogenic diets for MASLD rodent models [24–27] may result in adverse effects on liver health, body fat, insulin resistance and systemic inflammation [26, 28–32], we mainly focus on human studies in this review.

## Definitions and types of ketogenic diets

The term “ketogenic diet” was first proposed by Dr Wilder in the 1920s for treatment of epilepsy [33]. The classic ketogenic diet (CKD) for epilepsy consists of 90% fat with very low carbohydrates and no calorie restriction [34]. Since then, studies have explored the effects of ketogenic diets in many diseases with varied macronutrient composition and calorie limits. In view of the heterogeneity across studies, efforts have been made towards a universal definition and classification for ketogenic diets.

An expert consensus in 2024 on lower-carbohydrate diets defined very-low-carbohydrate, ketogenic diets as having a carbohydrate intake of 20–50 g/day, which corresponds to < 10% of total calorie intake if assuming an intake of 2000 kcal/day [35]. This definition aligns with a previous scientific statement by National Lipid Association on the effects of low- and very-low-carbohydrate diets for body weight and cardiometabolic risk factors. It is based on the typical maximum amount of carbohydrates allowed under ketosis, although the threshold is highly individualized and < 20 g/day may be needed for some people [36]. Moreover, it was recommended by the expert consensus to use absolute intake in grams instead of percentage to define ketogenic diets in research for better standardization [35]. Apart from carbohydrate restriction, it should be noted that ketogenic diets are not high in proteins, as excessive protein intake would increase gluconeogenesis from the remaining amino acids, which stimulates insulin secretion and thus interferes with ketogenesis. Protein intake in ketogenic diets should meet the recommended daily allowance (0.8 g/kg body weight per day [37]) and usually should not exceed 1.5 g/kg body weight per day [36].

A systematic review in 2020 summarized ketogenic diets into two categories: high-fat ketogenic diets and very-low-calorie ketogenic diets (VLCKD) [38]. The authors defined the former as diets with < 20–50 g/day carbohydrates and slightly more than sufficient proteins (0.8–1.2 g/kg body weight per day). Total calorie and fat intake can either be unrestricted or moderately restricted when aiming for weight loss. VLCKD also restrict the carbohydrate intake to < 20–50 g/day, but limit the total calorie intake to < 800 kcal/day, often with a higher amount of proteins (1.2–1.4 g/kg body weight per day) [38]. This is in line with the classification system proposed by Trimboli et al., which further divided high-fat ketogenic diets into isocaloric and low-calorie ketogenic diets [39] (Table 1).

**Table 1:** Types of ketogenic diets

Types of ketogenic diets	Composition			
	Total calorie (kcal/day)	Carbohydrates (g/day)	Proteins (g/kg body weight/day)	Fats*
Isocaloric (high-fat) ketogenic diets	Unrestricted	< 20–50^	0.8–1.2	Unrestricted
Low-calorie (high-fat) ketogenic diets	Moderately restricted	< 20–50^	0.8–1.2	Unrestricted
Very-low-calorie ketogenic diets	< 800 kcal/day	< 20–50	1.2–1.4	Restricted

^Corresponds to < 10% if assuming 2000 kcal/day

*Make up the remaining calorie intake after satisfying the requirements on carbohydrates and proteins

Besides macronutrient composition and total calorie intake, other dietary advice, such as the type of fats (monounsaturated fats [40, 41], essential fatty acids [40, 42], saturated fats [43, 44]), and nutritional supplements (vitamins, minerals, omega-3 fatty acid [41, 42, 45]) also vary among studies. For example, some proposed that a “well-formulated” ketogenic diet (WFKD) designed for long-term, sustained benefits should be composed of whole, unprocessed food, mainly saturated fats, adequate fluids and electrolytes with no calorie counting [46–48]. Some formulated a ketogenic Mediterranean diet with olive oil and omega-3 fish oil as the main source of fats [40, 49].

## Proposed mechanisms of ketogenic diets on MASLD

Ketogenic diets may benefit patients with MASLD by promoting weight loss, reducing inflammation and insulin resistance [50].

### Weight loss

Weight loss is an important strategy in MASLD management. It is generally agreed among major hepatology associations that a weight loss of 7–10% improves hepatic steatosis in patients with overweight and obesity, and > 10% weight loss can improve inflammation and fibrosis [19, 20, 51, 52]. A weight loss of 3–5% has even been suggested for lean patients [53]. The effectiveness of ketogenic diets in promoting weight loss is widely recognized [54]. Weight loss in ketogenic diets may be achieved without calorie counting. Unintentional decline of total calorie intake in ketogenic diets was observed in some studies [55, 56], which may be explained by higher satiety or suppressed appetite. It is recognized that proteins produce higher satiety effects and thermic response than carbohydrates and fats [57], and protein intake is usually slightly higher than the recommended daily allowance (0.8 g/kg body weight per day [37]) in ketogenic diets. A systematic review and meta-analysis of pre- vs post-test data from 12 studies found that individuals consuming ketogenic diets had reduced appetite despite calorie restriction [58]. Ketone bodies have been shown to suppress appetite by inducing changes in relevant hormones, such as ghrelin and glucagon-like peptide 1 [59, 60], although the range of plasma ketone levels required to achieve this effect is unclear. Even with similar calorie intake, a review of RCTs found ketogenic diets may lead to greater weight loss compared to other diets with higher carbohydrate intake due to greater reduction of body fat as it is utilized for fuel [61]. Moreover, some studies showed that patients with obesity who consumed a low carbohydrate diet (20% carbohydrates) had a small increase in their total energy expenditure compared to those with higher carbohydrate intake [62], but others obtained inconsistent results [63]. Thus, it remains controversial whether ketogenic diets lead to weight loss via increasing energy expenditure.

### Reduce inflammation

Increased local and systemic inflammation is critical in MASLD progression from simple steatosis to steatohepatitis, making the liver vulnerable to further damage [20, 64].

Ketogenic diets may reduce inflammation simply through their effects on weight loss, as weight loss is associated with reduction of pro-inflammatory cytokines in people who are overweight or obese [65]. Ketogenic diets also increase mitochondria efficiency in human skeletal muscle [66], as such they reduce inflammation related to mitochondrial dysfunction and oxidative stress [67].

Ketogenic diets may also reduce inflammation through the ketone metabolite beta-hydroxybutyrate (βHB). Pre-clinical and human studies showed βHB has potent anti-inflammatory properties by binding to the hydroxy-carboxylic acid receptor 2 on immune cells in various tissues, including the vascular and adipose tissue [68]. Also, βHB can inhibit NLRP3 (nucleotide-binding oligomerization domain-, leucine-rich repeat-, and pyrin domain-containing protein 3) inflammasome [69], a major contributor of inflammation in MASLD that facilitates the maturation of pro-inflammatory cytokines IL-1β and IL-18 [70]. Moreover, βHB can directly act as a scavenger for hydroxyl radicals [71], and increase the transcription of genes encoding oxidative stress resistance factors by epigenetic modification [72].

### Reduce insulin resistance

Insulin resistance is central in MASLD pathogenesis [11]. Ketogenic diets may reduce insulin resistance by promoting weight loss and reducing inflammation. Weight loss has been associated with improved insulin sensitivity by increasing adiponectin [73], while pro-inflammatory cytokines can impair insulin secretion in the β-cells of pancreatic islets and block insulin signaling in target tissues [74]. With a very low intake of carbohydrates, especially simple and refined ones, ketogenic diets may also reduce insulin resistance directly through improvements of both post-prandial surge [75] and the fasting levels [76] of glucose and insulin in patients with hyperglycemia and hyperinsulinemia. A low and stable insulin level reduces hepatic de novo lipogenesis and enhances fatty acid oxidation, thereby lowering liver fat content [77]. In addition, as ketogenic diets shift the metabolism to fuel from fat instead of from glucose, the loss of excessive body fat, especially ectopic fat in the muscle, liver and pancreas, sensitizes the cells to insulin signals and reduces systemic insulin resistance [78].

## Outcomes of ketogenic diets in patients with MASLD

### Outcomes on liver steatosis, MASH and fibrosis

Previous studies on the effects of ketogenic diets on liver fat content, MASH and fibrosis among patients with MASLD were mainly short-term [79, 80] or medium-term [40, 42–45, 81], with [43, 45, 79, 81] or without [40, 42, 44, 80] calorie restriction. The effects of ketogenetic diets compared to low-fat, high-carbohydrate (LFHC) diets on liver fat were also of research interest. Findings from these studies were summarized in the following.

#### Short-term studies

A ketogenic diet with calorie restriction (6% carbohydrates, 64% fats, 1000 kcal/day deficit) for only six days resulted in a 31% relative reduction from 10.3 ± 2.3% (mean ± standard deviation) to 7.1 ± 2.0% (*P* < 0.001) in intrahepatic triglycerides (IHTG) in ten patients with MASLD [79]. This reduction was attributed to increased hydrolysis of IHTG and the subsequent partitioning of fatty acids toward ketogenesis, as determined by positional isotopomer nuclear magnetic resonance tracer analysis. A ketogenic diet without calorie restriction (4% carbohydrates, 74% fats) for 2 weeks also resulted a significant 43.8% relative reduction in liver fat assessed by magnetic resonance spectroscopy in 17 patients with MASLD and obesity, with an unintentional slight weight loss of 1.8% [80]. This occurred alongside decreased expression of genes related to hepatic de novo lipogenesis.

#### Medium-term studies

A VLCKD (20–50 g/day carbohydrates, 1–1.4 g/kg/day proteins, 15–30 g/day fats, < 800 kcal/day) for eight weeks in 87 patients with overweight or obesity (90% with MASLD) reduced hepatic steatosis, as shown by controlled attenuation parameters dropping from 287 (255–325) to 230 (188–278) dB/m (*P* < 0.001) (81). There was also a small reduction in liver stiffness parameters among these patients (5.5 (4.3–6.5) versus 5.3 (4.0–6.5), *P* = 0.04). Another small study showed that a six-week ketogenic diet (8% carbohydrates, 70% fats, 15% calorie deficit) reduced IHTG among patients with obesity, especially those with liver steatosis [43]. A longer study of 6.5 months’ ketogenic diet aiming for 10% weight loss (on average 900-1100 kcal/day, ~ 50 g/day carbohydrates) led to reduced Fibrosis-4 index from 2.25 ± 0.23 to 1.40 ± 0.13 (P < 0.05) in patients with MASH [45]. Medium-term studies without calorie restriction also reported beneficial effects on patients with MASLD. A ketogenic Mediterranean diet rich in olive oil and omega-3 fish oil without calorie restriction (< 30 g/day carbohydrates) for 12 weeks reduced the steatosis degree by ultrasound in 14 patients with obesity and MASLD, with complete remission observed in 21.4% of the patients [40]. Tendler et al. conducted a pilot study using a ketogenic diet without calorie limit (< 20 g/day carbohydrates) for six months in five patients with biopsy-confirmed MASLD. They reported histological improvements in hepatic steatosis and necroinflammation in all but one patient who had poor dietary compliance [44].

#### Comparisons with LFHC diets

Some studies compared ketogenic diets to LFHC diets with similar calorie restriction. Kirk et al. conducted a randomized controlled trial (RCT) comparing the effects of a ketogenic diet (75% fats, 10% carbohydrates) with a LFHC diet (20% fats, 65% carbohydrates), both having 1000 kcal/day deficit, in 22 patients (half having MASLD) [82]. After 48 h, IHTG levels dropped more significantly in the ketogenic diet group (29.6 ± 4.8% vs 8.9 ± 1.4%, *P* < 0.05), but there was no difference between the two groups after ~ 11 weeks when both achieved 7% weight loss. Similarly, another study also showed no difference in liver fat reduction after a six-week ketogenic diet (70% fats, 9% carbohydrates) or LFHC (25% fats, 55% carbohydrates) with similar weight loss [43]. However, Browning et al.’s RCT in 18 patients for two weeks found greater reduction in IHTG in the ketogenic diet group (8% carbohydrates, 59% fats) (from 22 ± 10% to 10 ± 7%) compared to the low-calorie diet group (50% carbohydrates, 34% fats) (from 19 ± 10% to 14 ± 7%) after 2 weeks (*P* = 0.049), despite similar weight loss [83]. In another one-year non-randomized study, 262 patients with type 2 diabetes (> 90% with MASLD) followed a ketogenic diet without calorie restriction (< 30 g/day carbohydrates, 1.4 g/kg/day proteins). There was a significant reduction in the NAFLD liver fat score (− 1.95 ± 0.22, *P* < 0.001) and NAFLD fibrosis score (− 0.65 ± 0.06, *P* < 0.001), whereas the 87 patients who were suggested a LFHC diet had no change in NAFLD liver fat score (0.47 ± 0.41, *P* = 0.26) and a slight increase in NAFLD fibrosis score (0.26 ± 0.11, *P* = 0.02) with less weight loss [42].

Overall, these findings highlight the potential of ketogenic diets for improving hepatic steatosis, MASH, and fibrosis, which can be observed even after 1–2 week(s) of ketogenic diets. Improvements in liver steatosis can be observed with a ketogenic diet, even in the absence of significant weight loss. When compared to LFHC diets, calorie-matched ketogenic diets appear to be more efficient in reducing liver fat content in short term, but whether this is associated with greater weight loss remains uncertain.

### Outcomes on fasting lipid profile

Patients with MASLD often exhibit atherogenic lipid profiles associated with increased risk of CVD, including elevated plasma triglycerides (TG) and low-density lipoprotein cholesterol (LDL-c), and reduced high-density lipoprotein cholesterol (HDL-c) [84]. A recent review on adults with epilepsy who consumed ketogenic diets found about 12.9% reported elevated LDL-c [85]. The effects on lipid profile in patients with MASLD is of particular concern.

#### Fasting TG

Studies showed that short-term [41, 79, 80, 83] and medium-term (from six days up to a year) [40, 42, 45, 81, 86–88] ketogenic diets both reduced plasma TG in patients with MASLD, with [41, 45, 79, 81, 83, 86–88] or without [40, 42, 80] calorie restriction. Whether ketogenic diets are more effective in reducing TG than calorie-matched LFHC diets seems inconclusive. Browning et al.’s RCT in 18 patients showed no significant difference in TG between the two groups after two weeks [83]. Hu et al.’s RCT in 104 patients showed lower plasma TG, accompanied by greater weight loss, in the ketogenic diet group (from 3.39 ± 0.52 to 1.41 ± 0.32 mmol/L) compared to the usual care group (from 3.43 ± 0.59 to 1.75 ± 0.34 mmol/L) after three months (post-intervention *P* < 0.001) [88].

#### Fasting HDL-c

Results on the impact of ketogenic diets on plasma HDL-c were mixed. Two three-month RCTs, in 74 and 104 patients with MASLD, respectively, found a significant increase in HDL-c after hypocaloric ketogenic diets [87, 88], with no significant change [87] or less elevation [88] in the usual care group. A three-month single-arm study in 14 patients using a ketogenic Mediterranean diet rich in olive oil and omega-3 fish oil without calorie restriction (< 30 g/day carbohydrates) found an increase in HDL-c after the intervention (from 1.11 ± 0.03 to 1.52 ± 0.03 mmol/L) [40]. Another one-year non-randomized trial also found an increase HDL-c in the ketogenic diet group without calorie restriction, but not in the usual care group [42]. On the other hand, a reduction in HDL-c was found in two single-arm studies using calorie-restricted ketogenic diets for four [56] and eight weeks [81], respectively. A few other trials with different duration and calorie intakes did not identify any significant change in HDL-c levels [41, 44, 45, 79, 80, 86].

#### Fasting LDL-c

Results regarding the impact of ketogenic diets on plasma LDL-c were also inconsistent. Two single-arm studies using VLCKD for one to two months [41, 81] and another one using a ketogenic diet without calorie restriction for three months [40] reported a reduction of LDL-c in patients with MASLD. A 3-month RCT showed a greater reduction in LDL-c levels in those assigned to “ketogenic diet plus exercise” group (from 3.85 ± 0.83 to 1.50 ± 0.34 mmol/L) than those in the “exercise only” group (from 3.84 ± 0.29 to 2.50 ± 0.14 mmol/L) (post-intervention *P* < 0.001) [89]. Similarly, another 3-month RCT also reported a greater reduction in LDL-c levels in patients having a ketogenic diet compared with those having a LFHC diet [88]. Other studies of varied durations and calorie intakes reported no significant changes in LDL-c after ketogenic diets [44, 45, 55, 79, 80, 86, 87]. However, Vilar-Gomez et al.’s one-year non-randomized study on patients with type 2 diabetes (> 90% having MASLD) [42] found a 10% increase in plasma LDL-c in the ketogenic diet group [47]. The authors postulated that the elevated LDL-c was due to mobilization of adipose cholesterol stores and that it may not necessarily lead to higher risk of CVD because there was no change in apolipoprotein B concentration, which is a better measure of CVD risk than LDL-c [90]. Another study showed no difference in coronary plaque burden despite high LDL-c (> 4.9 mmol/L) after 4.7 years’ ketogenic diets in healthy individuals, compared to matched individuals with normal LDL-c [91].

Overall, current evidence suggested that ketogenic diets tend to reduce plasma TG in patients with MASLD, while the impacts on HDL-c and LDL-c remain uncertain.

### Outcomes on systemic inflammation

Low-grade chronic systemic inflammation is commonly seen in patients with MASLD [64]. Despite extensive pre-clinical studies on the use of ketogenic diets in alleviating inflammation in neurological diseases [92], limited studies investigated the effects of ketogenic diets on systemic inflammation in patients with MASLD. Mardinoglu et al. reported significant reduction in TNF-α and IL-6 after a two-week isocaloric ketogenic diet in ten patients with MASLD and obesity [80]. A study using VLCKD for eight weeks reported lower white blood cells and platelet counts in 87 patients with overweight/obesity (90% having MASLD) [81]. A one-year intervention showed reduced high-sensitivity C-reactive protein (hsCRP) levels in 262 patients (> 90% having MASLD) consuming a ketogenic diet without calorie restriction [42], but another study reported no change in CRP after 45-day VLCKD in 65 patients with MASLD [41]. Goss et al.’s RCT on 32 children and adolescents with MASLD and obesity found no significant changes in hsCRP in those consuming a isocaloric, low-carbohydrate (non-ketogenic) diet (< 25% carbohydrates, > 50% fat, 25% protein), and there was no difference when compared with those on a LFHC diet [93]. These findings aligned with the conclusion of a recent systematic review and meta-analysis of 44 RCTs (18 carried out in healthy individuals) on the effects of ketogenic diets on inflammatory markers in adults, which reported ketogenic diets lowered TNF-α and IL-6 levels compared with control groups, while the effects on CRP were uncertain [94]. Subgroup analyses found greater reduction in TNF-α in shorter-term studies (≤ 8 weeks) and greater reduction in IL-6 among those with higher body mass index (> 30 kg/m^2^).

Current limited evidence suggested that ketogenic diets may reduce TNF-α and IL-6 levels as well as white blood cell and platelet counts in patients with MASLD, while the effects on their CRP or hsCRP remain uncertain.

### Outcomes on gut microbiota

Studies investigating the effects of ketogenic diets on the gut microbiota of patients with MASLD are scarce. Mardinoglu et al. demonstrated that the reduced TNF-α and IL-6 after a two-week isocaloric ketogenic diet was associated with increased folate production by the gut microbiota, due to more growth of the folate-producing *Streptococcus* and *Lactococcus* [80] in ten patients with obesity and MASLD . Studies in other patients or healthy populations showed seemingly contradictory effects of ketogenic diets on gut microbiota. Beneficial changes with ketogenic diets, including reduction of pro-inflammatory bacteria (such as *Desulfovibrio* [95] and Proteobacteria [96]), and less Firmicutes with more Bacteroidetes [97, 98] which is associated with less energy extraction from food and less weight gain [99], have been reported. Meanwhile, unfavorable changes have also been reported, including reduced diversity [98, 100], and reduction of *Bifidobaceria* [101] which is generally associated with good health [102]. Low fiber intake is a concern of ketogenic diets. Short-chain fatty acids are produced by gut microbiota through fermentation of dietary fiber and have important functions in host health [37], including potential alleviation of liver steatosis [103]. Gardner et al.’s randomized crossover trial showed that participants had lower fiber intake when they were on a WFKD compared to when on a Mediterranean diet [104]. It has been suggested that the increased βHB level with ketogenic diets may exert some benefits of butyrate, the main short-chain fatty acid produced by gut microbiota, thereby reducing the need for dietary fiber. This is echoed by an RCT showing no additional benefits on weight loss, body composition and plasma lipid profile after adding soluble fiber to a ketogenic diet, except for greater reduction in LDL-c [105].

The effects of ketogenic diets on the gut microbiota and its metabolome of patients with MASLD are likely to be complex, and more studies are warranted to dissect them.

### Outcomes by types of fats in ketogenic diets

Fats are consumed in great amount in ketogenic diets, as such the types of fats may affect the risks and benefits of ketogenic diets. So far, a few related studies mainly compared saturated versus unsaturated fats, focusing on lipid profile and inflammatory markers.

A group of ketogenic diet advocates have proposed the consumption of mainly saturated fats [46–48]. Their group demonstrated that eight weight-stable men who had a ketogenic diet mainly composed of saturated fats (30.8% saturated fats) for six weeks had a slightly greater increase in LDL-c and less reduction in TG, compared to the levels when having a ketogenic diet mainly composed of unsaturated fats (17.0% saturated fats) [106]. However, the mean LDL-c particle size increased after both diets. The authors postulated that saturated fats consumed in ketogenic diets can be efficiently utilized, as no change was observed in plasma saturated fatty acids. On the other hand, some have proposed a ketogenic Mediterranean diet with unsaturated fats, such as olive oil and omega-3 fish oil being the main source of fats [40, 49, 107]. Addition of omega-3 supplements on top of a ketogenic diet for four weeks resulted in greater reduction in TG, IL-6 and IL-1β, with no difference in HDL-c or LDL-c in people who were overweight [108].

### Outcomes by genetic factors

Some SNPs have been associated with the response to ketogenic diets in terms of weight loss and body composition [109]. Genetic factors have moderate influence on food preferences, such as starchy foods or protein foods (meat or fish) [110, 111], which may be associated with individual acceptance of ketogenic diets. There is only one related study by Sevastianova et al. which showed that patients with MASLD who are homozygotes for rs738409 PNPLA3 G allele, which is associated with higher risk of MASLD independent of obesity [112], had more reduction in liver fat content after a six-day ketogenic diet (< 20 g/day carbohydrates, 1000 kcal/day deficit), compared to those who are homozygotes for the C allele (percentage change -45% vs -18%), despite similar liver fat content at baseline and weight loss during the intervention [113]. This indicates that the response to ketogenic diets may be personalized based on genetic background, highlighting a research gap in MASLD as to whether patients with certain genetic composition are more suitable to be managed by ketogenic diets.

## Sustainability

The sustainability of ketogenic diets has been questioned. The lack of long-term benefits of ketogenic diets in management of chronic diseases over two years [114] is potentially related to their low sustainability. Gardner et al. followed up 40 patients with prediabetes or type 2 diabetes for 12 weeks after they finished a randomized crossover trial that put them on either a Mediterranean diet or a WFKD, during which they followed their preferred dietary pattern. They found that patients’ diets were more similar to the Mediterranean diet than to the WFKD during this period, indicating that even a WFKD without calorie counting could be hard to sustain in a free-living environment [104]. Sustainability of a dietary pattern is complex and can be influenced by many individual, cultural and economic factors [115]. The requirement of very low carbohydrate intake in ketogenic diets goes against the current dietary norms where refined carbohydrates usually dominate the meals [116]. With more intake of animal proteins, ketogenic diets may be more costly than dietary patterns with higher carbohydrates and less protein foods [117]. The increased food costs present a challenge to patients with MASLD who are of lower socioeconomic status [118]. It has been suggested that clear guidance, self-monitoring (e.g., checking urinary/blood ketone) and social support may help improve sustainability among patients [115]. If ketogenic diets could not be sustained in the long term, rebound may be a problem if there is an abrupt switch to other dietary patterns with higher carbohydrate intake [119]. Multi-phase studies have shown that a transition that maintains the benefits obtained in ketogenic diets, e.g., a lower body weight, may be achieved by gradual reintroduction of carbohydrates with close monitoring by health professionals [120, 121].

## Side effects

The low sustainability of ketogenic diets may be related to the side effects. The most common short-term side effects observed among patients with MASLD and/or obesity are gastrointestinal distress (constipation, nausea, vomiting, diarrhea) and headache or dizziness [87, 107, 122–125]. Muscle cramping [44], weakness [107], sleep disturbance [107] and dehydration [124] have also been reported. These symptoms sometimes led to study drop-outs [87], but were generally mild and not clinically relevant. They may be attributed to insufficient keto-adaptation in short term [126]. A systematic review and meta-analysis of 16 studies on epilepsy showed that CKD (90% fats) induced more side effects than the modified Atkins diet (70% fats) and low-glycemic index diet (60% fats) [85], indicating that lower fat ratio may be better tolerated. Fluid and micronutrient intake may also play a role. Lower insulin levels in ketogenic diets may increase the excretion of sodium, potassium and water through the kidney [127], leading to electrolyte imbalance and dehydration. Refraining from whole grains, legumes and fruits in ketogenic diets may lead to insufficient intake of fiber and magnesium [104], which could be a reason for the gastrointestinal symptoms [128] and muscle cramping [129], respectively.

Some long-term concerns on ketogenic diets include kidney stones, bone loss and micronutrient deficiency [35]. A systematic review of 36 studies with a mean follow-up time of 3.7 years reported a 5.9% incidence of kidney stones in patients on ketogenic diets, of which 48.7% were uric stones, potentially related to a higher intake of protein and purine-rich foods (e.g., red meat, fish, poultry) [130]. However, few studies reported urate levels after ketogenic diets in patients with MASLD: only Rinaldi et al. reported no change in uric acid after an eight-week VLCKD in patients with MASLD [131]. On the other hand, a systematic review of seven clinical trials, ranging from three weeks to two years, reported no significant change in bone mass density after ketogenic diets [132]. Long-term nutrition adequacy may also be a concern for ketogenic diets. A systematic review revealed that without supplements, ketogenic diets for children with epilepsy were insufficient in most micronutrients [133]. However, ketogenic diets for metabolic diseases generally allow more carbohydrates and less fats than those for epilepsy. Kenig et al. found that the intake of ﻿magnesium, calcium, iron, phosphorus, and potassium was lower than the recommended levels during a 12-week ketogenic diet (75% fats, 5–10% carbohydrates) in people who are obese. Although their serum levels remained within the normal range, the serum calcium level decreased significantly ﻿(from 2.52 ± 0.10 to 2.36 ± 0.07 mmol/L) [134]. [Selection of nutrient-dense foods could potentially avoid nutrition deficiencies from long-term ketogenic diets [136].

Changes in thyroid hormones and acute pancreatitis after ketogenic diets have been reported. Iacovides et al. observed that after a three-week ketogenic diet (15% carbohydrates, 60% fats) in healthy participants, there was greater reduction in plasma triiodothyronine levels compared to when they were on a LFHC diet (55% carbohydrates, 20% fats), despite remaining in the normal range [137]. A case report presented a patient who suffered from acute pancreatitis without other established risk factors after having a “cyclic” ketogenic diet for three weeks, with ketogenic on weekdays and no restrictions on weekends. This aligned with a few other case reports, suggesting that significant alternation in pancreatic secretory activity attributed to dietary macronutrient composition may lower the threshold for acute pancreatitis [138]. More studies are needed to investigate the effects of ketogenic diets on thyroid and pancreas function and their indications on safety.

## Summary

With the change of nomenclature from NAFLD to MASLD, more attention has been given to the metabolic comorbidities associated with the disease, highlighting the need for treatments that address liver outcomes and related metabolic conditions from a holistic perspective. Based on existing literature, ketogenic diets, with or without calorie restriction, are beneficial for patients with MASLD in terms of reducing liver steatosis, MASH and fibrosis, as well as improving plasma TG in short and medium term. Ketogenic diets are more efficient than calorie-matched LFHC in reducing liver steatosis in the short term. Most short-term side effects are predictable and may be relieved by supplementation and well-formulated, nutrient-dense ketogenic diets. However, low sustainability and potential long-term health concerns present challenges to the long-term use of ketogenic diets in the management of MASLD and related chronic conditions.

Our review showed some key research gaps for future study. First, evidence on the impact of ketogenic diets on plasma HDL-c and LDL-c was mixed, as such the impact of ketogenic diets, both short-term and long-term, on CVD risk remain to be determined. Second, despite that reducing inflammation is proposed to be an important mechanism through which ketogenic diets improve liver health, few clinical trials have investigated changes in markers of systemic inflammation and the composition and metabolites of gut microbiota in patients with MASLD. Third, whether the consumption of saturated or unsaturated fats is more suitable for a ketogenic diet regime is still under debate. Importantly, the impacts of ketogenic diets on different conditions, such as lipid profile focusing on LDL-c particle number and size, systemic inflammation, and gut microbiome are significant considerations for formulating ketogenic diets that maximize and sustain benefits and minimize side effects.

## Data Availability

Data sharing not applicable to this article as no datasets were generated or analyzed during the current study.

## Abbreviations

MASLD: Metabolic dysfunction-associated steatotic liver disease
NAFLD: Non-alcoholic fatty liver disease
MASL: Metabolic dysfunction-associated steatotic liver
MASH: Metabolic dysfunction-associated steatohepatitis
CVD: Cardiovascular disease
TNF-α: Tumor necrosis factor-alpha
IL: Interleukin
CKD: Classic ketogenic diet
VLCKD: Very-low-calorie ketogenic diets
WFKD: “Well-formulated” ketogenic diet
βHB: Beta-hydroxybutyrate
NLRP3: Nucleotide-binding oligomerization domain-, leucine-rich repeat-, and pyrin domain-containing protein 3
ATP: Adenosine triphosphate
IHTG: Intrahepatic triglycerides
LFHC: Low fat, high carbohydrate
RCT: Randomized controlled trial
TG: Triglycerides
LDL-c: Low-density lipoprotein cholesterol
HDL-c: High-density lipoprotein cholesterol
hsCRP: High-sensitivity C-reactive protein
SNP: Single nucleotide polymorphism
PNPLA3: Patatin-like phospholipase domain-containing 3
